# No association between interleukin-18 levels and risk of cardiovascular disease: A Mendelian randomization study

**DOI:** 10.1186/s41065-020-00121-5

**Published:** 2020-04-07

**Authors:** Siyu Fan, Pan He, Jieqiong Guan, Wenjing Song, Hong Zhi, Lina Wang

**Affiliations:** 1 Key Laboratory of Environmental Medicine Engineering, Ministry of Education, Department of Epidemiology & Biostatistics, School of Public Health, Southeast University, 87 Ding Jiaqiao Rd, Nanjing, 210009 China; 2 Department of Cardiology, ZhongDa Hospital, Southeast University, Nanjing, China

**Keywords:** *IL-18* gene polymorphism, CVDs risk, Mendelian randomization, Meta-analysis

## Abstract

**Objective:**

In this study, Mendelian randomization method was used to determine whether there was a causal association between inflammatory cytokine IL-18 and cardiovascular disease risk.

**Methods:**

We performed a meta-analysis to evaluate the association between *IL-18*-137G/C and -607C/A polymorphisms and phenotype of IL-18 levels, and also the risks of CVD. All the literatures were searched before September 30, 2019. The logistic regression and linear regression were used to evaluate between IL-18 level and the risk of CVDs.

**Result:**

Twelve eligible articles of the association between *IL-18*-137G/C and CVD risks and 8 eligible literatures related to *IL-18*-607C/A and CVD risks; 2 qualified literatures of the association between *IL-18* SNPs and IL-18 levels and 4 eligible literatures related to IL-18 levels and CVD risks. Data of 4 literatures on the correlation between IL-18 level and CVD were summarized. Compared with patients with CVD, the mean of IL-18 level in the normal group was significantly decreased by 50.844 pg/ml (*P* < 0.05). But the association between *IL-18*-137G/C, *IL-18*-607C/A and CVD were not significant (*P* > 0.05), and the association between *IL-18*-607C/A and IL-18 level was also not significant (*P* > 0.05), Mendelian randomization study was failed to prove the association between IL-18 level and CVD risk.

**Conclusion:**

This study does not support a causal association between IL-18 level and the risks of CVD.

## Introduction

Cardiovascular disease (CVD) remains the leading cause of morbidity and mortality worldwide [1]. With an aging population and a rising burden of risk factors such as obesity and diabetes, it is projected that, by 2030, more than 23.6 million people around the world will die annually from acute myocardial infarction (MI), coronary artery disease (CAD), and other cardiovascular diseases [1]. The researchers suggested that CVD and its adverse consequence, myocardial infarction, were based on the inflammatory processes [2, 3]. Some inflammatory markers have been recently considered to be the diagnostic and prognostic biomarkers on CVD [4, 5].

Interleukin-18 (IL-18), of the cytokine family, is a proinflammatory cytokine which is expressed mainly by macrophages and acts on its receptor on the membrane of endothelial cells, lymphocytes, smooth muscle cells (all components of the atherosclerotic plaque) and induces Interferongamma (IFN-γ) production, endothelial dysfunction and plaque instability [6, 7]. Epidemiological studies have shown that the inflammatory factor IL-18 plays a very important role in the occurrence and development of cardiovascular diseases [8, 9]. The increased level of IL-18 was associated with the increased risk of CVD [10, 11]. However, some observational studies often overestimated or underestimated the causal relationships between the serum IL-18 level and risks of CVD because of the confounding factors including age, gender, smoking and drinking status, etc. [12].

The Mendelian randomization (MR) design adopt the concept of Instrumental Variable (IV) in econometrics and take genetic variants as IVs for the exposure factors to be studied. This method was used to determine whether the association between the exposure factors and the disease was causal [13]. Since alleles follow the Mendelian genetic rule of randomization allocation when gametes are formed, the association between genetic variants and disease risks is not distorted by the potential confounding factors and the reverse causal relationship in traditional epidemiological studies [14].

The *IL-18* gene locus is located at 11q22.2-q23.3and several polymorphisms in its promoter region have been identified [15]. Substitution of G > C at the location-137 changes a histone 4 transcription factor-1 (H4TF-1) nuclear factor-binding site, while a change of C > A at location-607 disrupts a cyclic adenosine monophosphate (cAMP) responsive element protein binding site. These changes might influence the transcriptional activity of the *IL-18* gene, and then associate with the change of the IL-18 level [15]. Some researchers suggested that the − 137 C-allele and the − 607 C-allele were associated with the higher IL-18 levels as compared to the − 137 G-allele and − 607 A-allele respectively [16, 17]. Therefore, we selected these two sites as IVs and, based on the principle of MR, using the meta-analysis method to construct the association between *IL -18* -137G/C (rs187238), −607C/A(rs1946518) polymorphisms and IL-18 level, *IL -18* -137G/C and -607C/A polymorphisms and risks of CVD, so as to infer the exact causal relationships between IL-18 level and CVD risks.

## Methods

### Publication search strategy

We used the following electronic databases to search studies: Pubmed, Web of Science, Chinese National Knowledge Infrastructure (CNKI) and Wanfang Data. Literature related to the association between *IL-18* gene variation and CVDs was comprehensively collected. The search was limited by date and extended from inception of each of these databases up until September 31, 2019. In addition, we manually searched reference lists of the eligible studies and recent reviews about this topic. The search terms and key words were (interleukin-18 OR interleukin 18 OR IL18 OR IL-18 OR IL 18) AND (CVDs and cardiovascular diseases OR heart OR coronary heart disease OR CHD) AND (level OR concentration OR polymorphism OR variant OR variation OR mutation OR SNP).

### Inclusion criteria and exclusion criteria

Inclusion criteria: (1) case-control study design;(2) the association between *IL -18* -137G /C or *IL -18* -607C /A polymorphisms and CAD risks or peripheral blood IL − 18 concentration should be studied and reported; (3) The correlation between peripheral blood IL-18 concentration and CAD risk should be studied and reported.

Exclusion criteria: (1) interventions such as drug treatment; (2) conference abstract or case report; (3) No control group or lack of cases.

### Data collection

Two investigators (SYF and PH) independently reviewed the titles, abstracts, and further full-text to determine whether include or not according to the inclusion and exclusion criteria. Relevant data were abstracted using a standard extraction form. The basic characteristics of each study should be extracted. Information was extracted according to the first author’s name, year of publication, type of cardiovascular disease, sample size, genotype count, the average level of IL-18 with different genotypes, and the *P* values of Hardy-Weinberg. Disagreements were resolved by the consensus of discussion.

### Quality assessment

The quality of the included literature was assessed using the NOS (Newcastle-Ottawa Scale) scale and star rating was made. The score range was from 1 to 9, with a score of 9 as the highest quality.

### Mendelian randomization

**Fig. 1:**
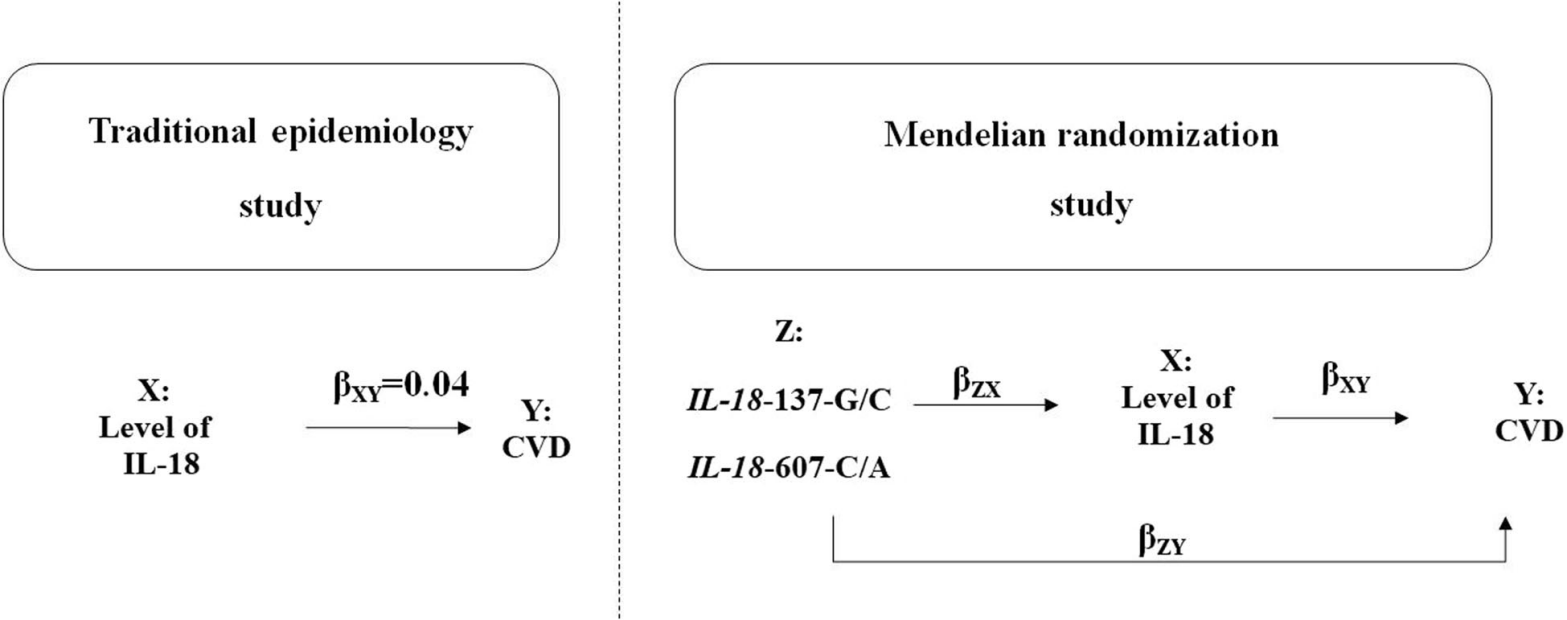
Mendelian randomized study design for the association between IL-18 level and CVD

As shown in Fig. 1, this mendelian randomization study was performed by introducing two genetic mutation sites of the *IL-18* -137G / C and -607C / A as instrumental variables Z. And we assumed X, Y to be IL-18, CVD, respectively. If the outcome is a continuous variable (such as IL18 level), use a linear regression model and the outcome is a binary variable (such as CVD or not), then use a logistic regression model (β = ln OR).

### Statistical analysis

Hardy -- Weinberg equilibrium test of the control group was performed using chi-square test (*P* < 0.05 was considered statistically significant, that is, the samples were not from the unified genetic population);

Statistical heterogeneity between studies was evaluated by I-square statistical test. According to the heterogeneity, fixed effect model or random effect model were used for combined effect analysis. If there was significant heterogeneity between studies (I^2^ > 50%), the random effect model was used for meta-analysis. Otherwise, fixed effect model was adopted. Risk estimates for genotype associated with CVDs was described by OR values and their corresponding 95% confidence intervals, then a logistic regression model was used to obtain the correlation between these two sites and the risk of cardiovascular disease (β_ZX=_ ln OR). The changes in IL-18 levels among genotypes were calculated by linear regression. The indirect correlation between IL-18 level and cardiovascular disease risk was β_XY_ = β_ZX_ / β_ZY_.

All statistical analysis was performed using the STATA 12.0 software.

## Results

### Literature search process

**Fig. 2:**
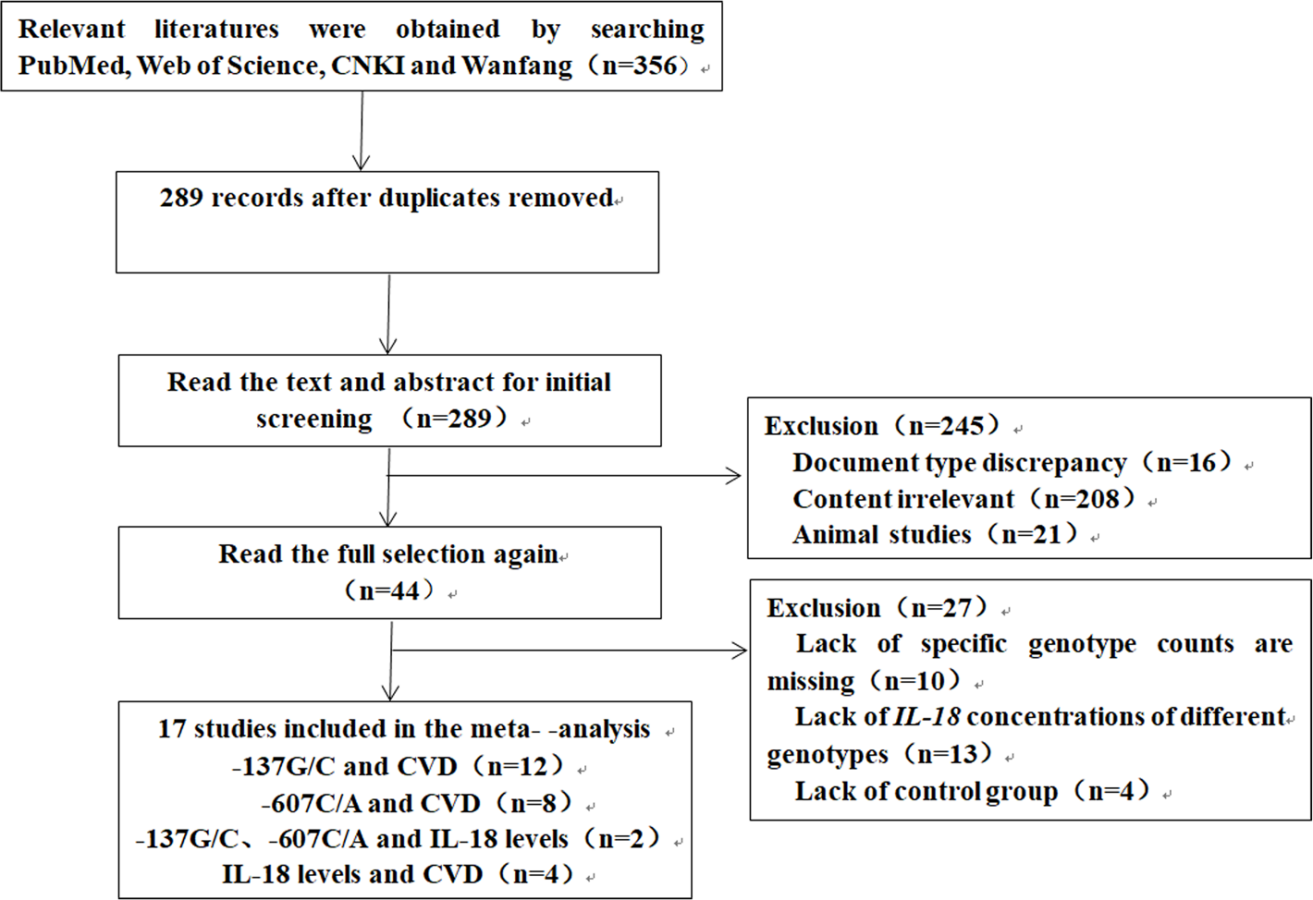
Flow chart of literature screening

After initial retrieval, a total of 356 potentially relevant literatures were obtained, and 18 qualified literatures were finally included in the study after screening. The literature screening process and results are shown in Fig. 2.

### Basic characteristics of of each included study

Totally, 18 papers were included, 12 were related to *IL-18* -137G/C and CVD risks, 8 articles on the association between *IL-18* -607 C/A and CVD risks; 2 were related to *IL-18* -137G/C、*IL-18* -607C/A and IL-18 level, and 4 articles on the association between IL-18 level and CVD risks. The included literatures involved 3 types of cardiovascular diseases, including coronary artery disease, premature coronary artery disease, myocardial infarction, etc.

The results of NOS scoring showed that the average score of the included literatures was 6.4, which indicated that the quality of the included literatures in this study was relatively high on the whole. (Tables 1, 2 and 3).

**Table 1:** Basic characteristics of the included literature (IL-18 level and cardiovascular disease risk)

Author	year	CVDs type	Sample size		IL-18level(pg/ml)		male(%)		average age		NOS score
			Case	Control	Case	Control	Case	Control	Case	Control	
Mallat [18]	2002	MI	42	11	196.50 ± 126.15	48.55 ± 10.670	29 (69.0)	7 (63.6)	62.6 ± 1.9	36.1 ± 4.0	7
Wang [19]	2006	CAD	45	30	373.60 ± 176.70	226.30 ± 88.90					5
Jefferis [20]	2013	MI	304	706	418.25 ± 37.80	387.02 ± 131.75	267 (73.4)	512 (72.5)	70.87 ± 5.47	70.83 ± 5.47	7
Blankenberg [21]	2003	CAD	335	670	225.12 ± 4.36	203.91 ± 2.88			55.3 ± 0.1	55.2 ± 0.1	7

*CVDs/CVD* cardiovascular diseases, *CAD* coronary artery disease, *MI* myocardial infarction, *ACS* acute coronary syndrome

NOS score: Newcastle-Ottawa Scale for Assessing the Quality of Nonrandomized Studies in Meta-Analysis, from 1 to 9

**Table 2:** Basic characteristics of the included literature (IL 18 genetic variation and IL-18 level)

Author	year	Sample size			*IL-18* level(pg/ml)-137 G/C			Sample size			*IL-18* level(pg/ml)-607 C/A			NOS score
					(mean ± SD)						(mean ± SD)			
		GG	GC	CC	GG	GC	CC	CC	CA	AA	CC	CA	AA	
Opstad [16]	2011	532	394	69	248.44 ± 20.54	245.41 ± 20.84	271.43 ± 37.22	364	500	132	243.39 ± 21.02	249.42 ± 20.34	253.33 ± 32.37	7
Mitrokhin [17]	2018	86	76	14	145.40 ± 22.09	94.44 ± 14.32	107.18 ± 23.72	56	90	28	117.08 ± 20.12	86.00 ± 11.58	106.96 ± 20.13	7

NOS score: Newcastle-Ottawa Scale for Assessing the Quality of Nonrandomized Studies in Meta-Analysis, from 1 to 9

**Table 3:** Basic characteristics of the included literature (*IL-18* -137G/C, 607A/C and CVD risks)

Author	year	CVDs type	Sample size		*IL-18* -137G/C			P_HWE_	*IL-18* gene -607A/C			P_HWE_	NOS score
					GG	GC	CC		AA	AC	CC		
			Case	Control	Case/Control				Case/Control				
Opstad [16]	2011	CAD	1001	240	532/108	394/82	69/14	0.788	364/74	500/96	132/34	0.762	7
Mitrokhin [17]	2018	CAD	176	116	86/52	76/48	14/12	0.853	56/28	90/66	28/20	0.081	8
Buraczynska [22]	2016	CVD	1103	445	439/214	562/212	102/19	0.002					5
Zhang [23]	2011	MI	468	432	352/308	112/116	4/8	0.439	170/90	210/220	88/122	0.616	7
Pei [24]	2009	MI	234	216	180/150	53/63	1/3	0.203	82/42	107/108	45/66	0.854	6
Kariž [25]	2013	MI	169	326	90/162	71/141	8/23	0.301	55/109	86/158	28/59	0.895	7
Rajesh [26]	2015	CAD	300	300	168/176	102/105	30/19	0.533					6
Hoseini [27]	2018	CAD	100	100	57/48	39/46	4/6	0.242					7
Liu [28]	2009	CAD	241	145	195/99	45/44	1/2	< 0.001					6
Jabir [29]	2017	CAD	98	99	48/49	19/16	9/9	< 0.001	65/59	0/2	9/13	< 0.001	6
Omer [30]	2016	PCAD	340	310	217/183	116/108	7/19	0.568					6
Deser [31]	2016	PCAD	55	61	36/35	18/22	1/4	0.83	72/46	110/78	55/35	0.72	5
Zhu [32]	2011	CAD	141	240					47/51	71/123	23/66	0.653	7

*CVDs/CVD* cardiovascular diseases, *CAD* coronary artery disease, *MI* myocardial infarction, *PCAD* premature coronary artery

NOS score: Newcastle-Ottawa Scale for Assessing the Quality of Nonrandomized Studies in Meta-Analysis, from 1 to 9

### Meta-analysis on the IL-18 levels and CVD risks

A total of 4 literatures related to IL-18 level and CVD risk were included, including 786 patients in the case group and 1417 normal people in the control group (Table 1). Compared with patients with CVD, IL-18 level in the control group was significantly decreased by 50.844 pg/ml (95%CI =33.455–68.233) (Table 4).

**Table 4:** Results of meta analysis on the association between IL-18 gene, IL-18 level and CAD

	Number of included references	Total number of participants	Case	Control	Heterogeneity		Effect size	
					*I*^*2*^	*P* values	OR/WMD	*P values*
-137G/C and CVDs	12	4273	2596	1677	74.60%	< 0.05	0.722 (0.440, 1.183)	> 0.05
-607C/A and CVDs	8	2202	1319	883	82.3%	< 0.05	0.726 (0.432, 1.218)	> 0.05
Level of IL-18 and CVDs	4	2203	786	1417	96.1%	< 0.05	50.844 (33.455, 68.233)	< 0.05

50.844 pg/ml (95%CI =33.455–68.233

### Association between IL-18 genetic variation and IL-18 levels

Two articles were included about IL-18 genetic variation and IL-18 levels (Table 2). The association between SNP and IL-18 levels was calculated using linear regression. The mean level of IL 18 among IL-18-137 CC carrier was significantly increased by 9.629 pg/ml compared to GG carriers (β_ZX1_ = 9.629 pg/ml, 95% CI =0.524–18.733). The mean IL-18 level of IL-18 -607 AA genotype carriers was decreased by 1.165 pg/ml compared with CC genotype carriers, and the 95% confidence interval was (β_ZX2_ = − 1.165 pg/ml, 95%CI = -9.716–7.385), but this association was not statistically significant.

### Association between genetic variation and CVD risks

A total of 12 studys related to IL-18-137G/C and CVD risks were included, including 2596 patients in the case group and 1677 normal people in the control group (Table 3). Due to the high heterogeneity between studies associated with IL-18-137G/C and CVD risk (I^2^ = 74.6% > 50%), the effect size was combined using a random effect model. Compared with genotype GG, genotype CC was associated with about a 27.8%(OR = 0.722, 95%CI = 0.4440, 1.183) lower risk of CVD. IL-18 -607C/A is another locus that has been intensively studied to affect IL − 18 level. A total of 8 articles were included, including 1319 patients in the case group and 883 normal people in the control group (Table 3). The random effect model was used to merge the effect quantities(I^2^ = 82.3% > 50%). Compared with genotype AA, genotype CC were 27.4%(OR = 0.726, 95%CI = 0.432, 1.218) less likely to develop CVD. But those association were not significant (Table 4).

The association between *IL-18*-137 GG genotype and CVD risk was not statistically significant, with a regression coefficient β_ZY1_ = − 0.326 (95% CI = -0.821–0.168). *IL-18*-607 AA genotype was also not significantly associated with CVD risk, with a regression coefficient β_ZY2_ = − 0.320 and a 95% confidence interval was (− 0.839–0.197).

### MR study on the association between IL-18 levels and CVD risks

While traditional epidemiological studies directly sought the association between IL-18 level and CVD risks, mendelian randomization is a method applied IVs to verify the causal relationship between exposures and diseases [13]. As shown in Fig. 1, we assumed X, Y, and Z to be IL-18 level, CVD risks, and IVs, respectively. And the Wald ratio (β_XY_) of IL-18 to CVD through a specified variant can be calculated as follows:

$$ {\upbeta}_{\mathrm{XY}}={\upbeta}_{\mathrm{ZY}}/{\upbeta}_{\mathrm{ZX}}, $$

$$ {\upbeta}_{\mathrm{XY}1}={\upbeta}_{\mathrm{ZY}1}/{\upbeta}_{\mathrm{ZX}1}=-0.034, $$

$$ {\upbeta}_{\mathrm{XY}2}={\upbeta}_{\mathrm{ZY}2}/{\upbeta}_{\mathrm{ZX}2}=-0.275 $$

But according to the association between IL-18 genes and IL18 level, the association between IL-18 genes and CVD risks, β_ZY1、_β_ZY2_ and β_ZX2_ were not statistical significance. A causal association between IL-18 levels and CVD risk could not be established.

Therefore, the current evidence does not support the association between *IL-18-137*G/C、*IL-18-607*C/A and CVD risks, nor does it support the association between *IL-18-607*C/A and IL-18 levels. Mendelian randomization was subsequently unable to confirm whether the association between IL-18 levels and cardiovascular disease risk is a causal association.

## Discussion

Current research suggests that CVD is a chronic inflammatory disease. Mallat et al. [33] studied carotid intima tissue in atherosclerotic patients and found that the expression of IL-18 in plaques was higher than that in normal segments, and the expression of IL-18 mRNA in unstable plaques was significantly higher than that in stable plaques. It was first proposed that IL-18 may have plaque instability, and this effect is thought to influence the plaque destabilization of extracellular matrix metalloproteinase inducer in subsequent studies. Recent studies have shown that it can delay the progression and stabilization of existing ang II -dependent vulnerable lesions by reducing IL-18-associated inflammation [34]. In this Mendelian randomization study, we used genetic variants as IVs for IL-18 and examined associations between IL-18 and risk of CVD.

Strengths of the current study include the high quality of the case-control and cohort studies used. The majority of the studies were population-based in design. Results from traditional observational epidemiological studies have shown a significant association between IL − 18 levels and CVD risk [35], but this conclusion has not been confirmed. Compared to results from traditional analyses of observational studies, the risk estimates using instrumental variables are less likely to be affected by confounding and other types of bias. We used SNPs known to be associated with IL-18 level, all located in or near genes encoding enzymes and carrier proteins involved in IL-18 synthesis or metabolism, which provided a strong and valid instrumental variable for IL-18 level.

In our study, we found that the subjects carried with *IL-18* -137GG genotype have significantly lower levels of IL-18 than that of CC carriers. But according to our summary results, the current evidence did not support the association between *IL-18*-137G/C,-607C/A and CVD risks, nor did it support the association between *IL-18*-607C/A and IL-18 levels. Mendelian randomization research was subsequently unable to confirm that genetically-elevated IL-18 level causally related with CVD risks.

There are several limitations in this study: Firstly, in this study, only *IL-18-137*G/C and *IL-18-607* C/A were selected as IVs for Mendelian randomization study, which was inadequately powered to determine a true association between the IL-18 polymorphisms and cardiovascular disease. Future studies can also include more loci to verify the association of IL18 levels with CVD. Secondly, the *IL-18*-137G/C and *IL-18*-607 C/A of *IL-18* we focused on in this study did not have a significant effect on the risk of CVD, the association between IL-18-607 C/A and IL-18 level was not statistically significant, that is, they were not the determining genes but the microgenes. This also showed that the selection of appropriate instrumental variables in Mendelian randomization studies was very important, and the improper selection may make the results difficult to explain. In the future, it is necessary to refer to the results of large GWAS studies to screen genes with more significant phenotypic effects for MR analysis. Thirdly, part of the included studies were hospital-based control group studies, which resulted in a lot of confounding. It may be the presence of these confounding factors that led to false negative results in the study on the association between *IL-18*-137G/C, IL-18-607 C/A and CVD risk. Fourthly, the heterogeneity of the association analysis between the *IL-18* genetic variants and the risk of CVD is relative in the high level, which might be caused by the races, because the distributions of the average allele frequency were different between the European and Asian population. Since only two papers about IL 18 genetic variation associated with level of IL 18 gene were included in this study, subgroup analysis was not conducted according to race. In addition, MR approach has its own limitations including linkage disequilibrium [36]. There are other genes in the vicinity of *IL-18*-607 C/A that also affect IL-18 levels, and our study did not exclude the influence of these potential genes, so these genes may become confounders to IL-18 levels. Because of the lack of research on these genes, such confusions are difficult to eliminate.

To sum up, this study provides evidence for Mendelian randomization studies on the association between IL-18 levels and CVD risk. Although no statistically different genotype-phenotype and genotype-disease data were obtained, it was ultimately impossible to support a causal association between IL-18 levels and CVD risk through MR method, it still provided new data and basis for relevant studies.

## Data Availability

The data used to support the findings of this study are included within the article.

## Abbreviations

ACS: Acute coronary syndrome
AF: Atrial fibrillation
CAD: Coronary artery disease
CVDs/CVD: Cardiovascular disease
IFN-γ: Interferongamma
IL-18: Interleukin 18
IV: Instrumental Variable
MI: Myocardial Infarction
MR: Mendelian randomization
PCAD: Premature coronary artery disease
SNP: Single nucleotide polymorphism
